# Facilitation of corticospinal excitability by virtual reality exercise following anodal transcranial direct current stimulation in healthy volunteers and subacute stroke subjects

**DOI:** 10.1186/1743-0003-11-124

**Published:** 2014-08-18

**Authors:** Yeun Joon Kim, Jeonghun Ku, Sangwoo Cho, Hyun Jung Kim, Yun Kyung Cho, Teo Lim, Youn Joo Kang

**Affiliations:** Department of Rehabilitation Medicine, Deahan Hospital, Suyudong, Gangbukgu, Seoul South Korea; Department of Biomedical Engineering, Keimyung University, Daegu, South Korea; The Graduate School of Technology & Innovation Management, Hanyang University, Seoul, South Korea; Department of Rehabilitation Medicine, Eulji Hospital, Eulji University School of Medicine, Hagye dong, Nowongu, Seoul 139-711 South Korea

**Keywords:** Virtual reality, Transcranial direct current stimulation, Subacute stroke, Upper extremity, Transcranial magnetic stimulation

## Abstract

**Background:**

There is growing evidence that the combination of non-invasive brain stimulation and motor skill training is an effective new treatment option in neurorehabilitation. We investigated the beneficial effects of the application of transcranial direct current stimulation (tDCS) combined with virtual reality (VR) motor training.

**Methods:**

In total, 15 healthy, right-handed volunteers and 15 patients with stroke in the subacute stage participated. Four different conditions (A: active wrist exercise, B: VR wrist exercise, C: VR wrist exercise following anodal tDCS (1 mV, 20 min) on the left (healthy volunteer) or affected (stroke patient) primary motor cortex, and D: anodal tDCS without exercise) were provided in random order on separate days. We compared during and post-exercise corticospinal excitability under different conditions in healthy volunteers (A, B, C, D) and stroke patients (B, C, D) by measuring the changes in amplitudes of motor evoked potentials in the extensor carpi radialis muscle, elicited with single-pulse transcranial magnetic stimulation. For statistical analyses, a linear mixed model for a repeated-measures covariance pattern model with unstructured covariance within groups (healthy or stroke groups) was used.

**Results:**

The VR wrist exercise (B) facilitated post-exercise corticospinal excitability more than the active wrist exercise (A) or anodal tDCS without exercise (D) in healthy volunteers. Moreover, the post-exercise corticospinal facilitation after tDCS and VR exercise (C) was greater and was sustained for 20 min after exercise versus the other conditions in healthy volunteers (A, B, D) and in subacute stroke patients (B, D).

**Conclusions:**

The combined effect of VR motor training following tDCS was synergistic and short-term corticospinal facilitation was superior to the application of VR training, active motor training, or tDCS without exercise condition. These results support the concept of combining brain stimulation with VR motor training to promote recovery after a stroke.

**Electronic supplementary material:**

The online version of this article (doi:10.1186/1743-0003-11-124) contains supplementary material, which is available to authorized users.

## Background

More than half of stroke survivors experience long-term upper extremity impairment, and this can significantly impact disability and general health after a stroke [1]. Given that there is no universally accepted treatment after stroke, studies on the development of new effective therapeutic strategies for upper extremity motor therapy, and on how recovery can be achieved most effectively after stroke are important [2, 3]. Recently, non-invasive brain stimulation, robotics, virtual reality (VR), and functional electrical stimulation have been developed for stroke rehabilitation [4]. Furthermore, there is growing evidence that a combination of non-invasive brain stimulation and motor skill training is a new treatment option in the field of neurorehabilitation [5, 6]. These newer combinations and training approaches are based on an increased understanding of the plasticity of the nervous system and how this plasticity facilitates motor learning, as influenced by frequency of use, skill development, and practice parameters [7, 8].

VR applications are relatively novel and potentially useful techniques in upper extremity rehabilitation after strokes. Moreover, interface technologies, augmented reality technologies, and various sensorimotor feedback techniques are rapidly advancing [9, 10]. Previously, there was limited evidence that the use of VR and interactive video gaming actually improved arm function, because there were few commercial devices and a lack of studies in the literature [11]. However, there is growing evidence regarding the effectiveness of upper extremity VR training compared to conventional therapies after stroke [12–14]. Recent experimental evidence suggests that corticospinal excitability is enhanced after VR-induced visuomotor learning conditions, and that VR technologies have great potential for the development of novel strategies for sensorimotor training in neurorehabilitation [15, 16].

Non-invasive methods of brain stimulation, including transcranial direct current stimulation (tDCS) and repeated transcranial magnetic stimulation (rTMS), are emerging techniques and have been found useful in facilitating recovery after various neurological disorders. tDCS applied at rest over the primary motor cortex (M1) can raise corticomotor excitability and transiently improve motor function in healthy participants and chronic stroke patients [17–21]. tDCS can be applied more readily than rTMS, which has been shown to enhance arm function and working memory, and to facilitate visual-spatial attention in stroke patients [22, 23].

We hypothesized that the combined effects of VR motor training following tDCS would be synergistic and their corticospinal facilitation would be superior to the application of VR training, active motor training, or tDCS alone, without exercise. We expected that post-exercise corticospinal facilitation would be higher and sustained longer after VR wrist exercises following tDCS than under other conditions. We used the TMS single-pulse paradigm because it is advantageous in identifying corticospinal excitability, according to various experimental conditions [24].

## Methods

### Participants

In total, 15 healthy, right-handed volunteers (13 males, 2 females), and 15 subacute stroke patients (11 males, 4 females) participated. All participants gave informed consent to participate and were educated about the experimental protocol, including the TMS procedure. The TMS procedure was approved by the institutional review board at our hospital.

The mean ages of the groups were 32.6 ± 8.9 (23-42) years for the healthy volunteers and 55.4 ± 17.6 (38-74) years for the stroke patients. While the mean ages of the two groups differed, the excitability and plasticity of the corticospinal system in response to motor activity or training were not expected to be affected by age [25–27].

The patients had sustained a primary ischemic or hemorrhagic stroke, as diagnosed by magnetic resonance imaging image scans or computed tomography. They presented with mild paresis of the upper extremity and lacked any additional neurological disease causing motor deficits. The motor scores by Medical Research Council (MRC) motor scales of affected wrist extension were above 3 and the Fugl-Meyer upper extremity assessment scale (FMS) was 55.3 ± 3.14 (50-62). A summary of demographic variables and clinical measure for the stroke group is included in Table 1.

**Table 1:** **Clinical and demographic characteristics**

Patient	Sex	Age	Weeks since onset	Etiology	Site of lesion	FIM	MBI	FMS (upper extremity)
1	M	38	5	Infarction	Cerebellum, pons	111	60	52
2	M	74	3	Infarction	Lt. pons	106	59	53
3	M	52	4	Infarction	Lt. MCA (subcortical)	101	97	55
4	M	66	6	Hemorrhage	Rt. BG (subcortical)	103	81	53
5	M	65	5	Infarction	Rt. pons	106	80	60
6	M	54	7	Hemorrhage	Lt. BG (subcortical)	99	83	56
7	F	74	5	Infarction	Lt. PICA (subcortical)	111	81	54
8	M	71	6	Hemorrhage	Rt. BG (subcortical)	102	74	50
9	F	47	8	Infarction	Rt. ACA (cortical)	106	82	62
10	F	56	4	Hemorrhage	Lt. MCA (cortical)	105	74	54
11	M	59	5	Infarction	Rt. BG (subcortical)	112	89	58
12	M	52	3	Infarction	Rt. MCA (subcortical)	106	81	58
13	F	62	5	Hemorrhage	Lt. MCA (subcortical)	108	76	56
14	M	57	4	Infarction	Rt. MCA (cortical)	122	88	57
15	M	64	4	Hemorrhage	Lt. BG (subcortical)	116	83	55

Rt, right; Lt, left; MCA, middle cerebral artery; BG, basal ganglia; PICA, posterior inferior cerebellar artery; ACA, anterior cerebral artery; FIM, functional independence measure; MBI, modified barthel index; FMS, Fugl-Meyer upper extremity assessment score.

Exclusion criteria were: (1) severe motor deficit (MRC score of wrist extension ≤ 2); (2) no motor evoked potential (MEP) in the affected extensor carpi radialis (ECR) muscle; (3) severe cognitive deficit with a score < 24 on the Mini-Mental State Examination [28]; (4) visual or hearing impairment or both, unilateral neglect or visual field deficits [29]; (5) those believed to give unreliable responses because of severe depression [30]; (6) contraindications for TMS (metallic implants or pacemakers) or tDCS (intracranial or orbital metallic implant); (7) previous symptoms of simulation sickness syndrome after VR exercise; (8) previous seizure history; (9) concurrent use of Na^+^ channel blocking agent or N-methyl-D-aspartate receptor antagonist, which might decrease the effects of anodal stimulation [31]; and (10) acute stroke (within 2 weeks).

### Experimental design

Four different conditions were provided in random order on separate days within four days. Condition A was an active wrist exercise program. Condition B was a VR wrist exercise program. Subjects played a computerized VR ski game for 15 min. In condition C, VR wrist exercise (as in condition B) followed anodal tDCS (anodal tDCS was delivered to the scalp of each subject over M1 of the non-dominant hemisphere in healthy volunteers and the affected hemisphere in stroke patients for 20 min). In condition D, anodal tDCS was performed without exercise; anodal tDCS was performed in the same manner as in condition C. Previous studies showed that facilitation of MEPs after single anodal tDCS for 13 min could be sustained more than 90 min post-stimulation [32]; as a result, different conditions were applied on separate days and the order was randomized across subjects using a computer-generated randomization list. In stroke patients, we studied the B, C, and D conditions.

### Active wrist exercise program

#### Set-up

Subjects were seated in a comfortable chair with armrests. Their shoulder and elbow joints were placed on the desk, and the experimenter instructed, monitored, and confirmed that there was only wrist movement during the experiment. The marker was located on top of the cylinder grasped by the user’s hand. All subjects grasped the cylinder-like interface with markers in a pattern. A camera (Webcam pro 9000, Logitech Inc., Romanel-sur-Morges, Switzerland) was used to calculate the 3D position and orientation of the marker with specific patterns.

#### Description of training activity

Each subject conducted routine repetitions of a full range of simple rhythmic wrist flexion and extension exercises using their left (healthy volunteer) or affected (stroke) wrist at the rate of 10/min, as indicated by a metronome. The subjects’ performance and attention during exercise were monitored by the experimenter. After a short familiarization session, one session of repetitive active wrist flexion and extension exercise were required for 15 min, and the subjects exercised while watching a black screen.

#### Performance metrics

Several parameters were obtained using a camera while the subject performed the exercises. Task speed was defined as angular velocity, and was calculated as the moving angle per second; average angular velocity was determined. Distance was defined as the angle between wrist flexion and extension; the average distance was determined. There were two steps for extracting the angles of wrist flexion and extension. In the calibration step, before the game started, it was confirmed that the user’s wrist was in the correct pose to give a specific position and orientation. Thus, once the initial position and orientation were confirmed in the initial pose, in the angle extraction step, the angle of the wrist extension and flexion could be calculated based on the calibration. The angles were validated in our experimental setup and errors were below 2°, which seemed negligible for playing the game.

### VR wrist exercise program

#### Set-up

**Figure 1:**
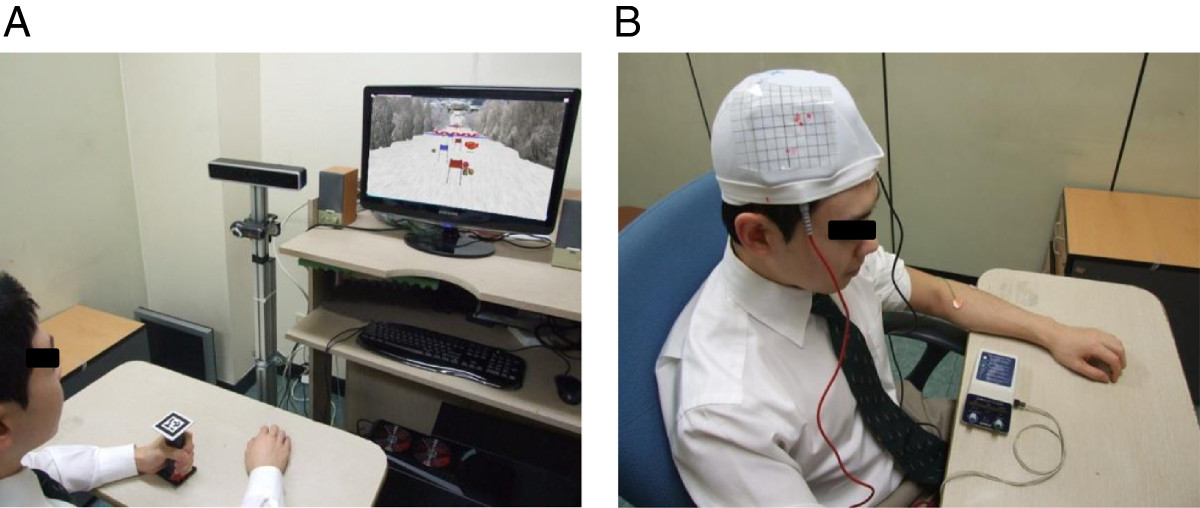
**Experimental set-up with a virtual reality exercise program (A) and transcranial direct current stimulation (B).**

The VR ski game for wrist exercise by stroke patients was developed by clinicians, occupational therapists, biomedical engineers, and software engineers. The software (Ski game for wrist exercise following stroke, Windows 7 environment, Metasio Asia Inc., Kyungki-do, Korea) was operated based on a computer vision technique using a camera and marker in a pattern, as described in the active wrist exercise session. The wrist exercise was selected because it plays a major functional role in complex movements of the upper extremity, such as hand shaping and grasping. Subjects were seated in a comfortable chair with armrests. Their shoulder and elbow joints rested on the desk, and the experimenter instructed, monitored, and confirmed, that there was only wrist movement during the experiment. All subjects grasped the cylinder-like interface with markers in a pattern (Figure 1-A) that were made for the study.

#### Descriptions of training activity

When subjects moved their wrists (extension, flexion), the computer-connected web camera recognized this movement and directed the movements of the virtual skier, producing a side-to-side turning motion in the overall downhill movement (rhythmic wrist flexion and extension) at a rate of 10/min (Figure 1-A). On the game screen, there were some coins in the corners (right and left sides), and the skier could acquire the coins via right and left turns. The subjects performed one session for 15 min in each of the conditions after a short familiarization session. The subjects’ performance and attention during exercise were monitored by the experimenter.

#### Performance metrics

The calibration and angle extraction steps were the same as in the active wrist exercise session. Task speed and distance were measured using a computer-connected webcam and marker. Motion was calculated at 30 Hz, which was sufficient to detect wrist movements for this study. The mean number of achieved coin per one minute and rate of successful coin acquisition in each game were calculated. The rates of coin acquisition were compared between conditions B and C, in which the VR exercise was conducted.

### tDCS

An anodal tDCS (Phoresor II PM850; Iomed Inc., Salt Lake City, UT, USA) was applied at the motor hot spot of the ECR muscle (where stimulus-evoked MEPs had the largest peak-to peak amplitudes) for 20 min (1 mA, Figure 1-B). For anodal stimulation, the anode (saline-soaked electrode, 5 × 5 cm) was placed over the M1 area of the non-dominant hemisphere in healthy volunteers and the affected hemisphere in stroke patients and the same size of cathode was placed over the contralateral supraorbital area. This stimulation duration and method have been commonly used in studies of motor learning [20, 33–35].

### TMS

**Figure 2:**
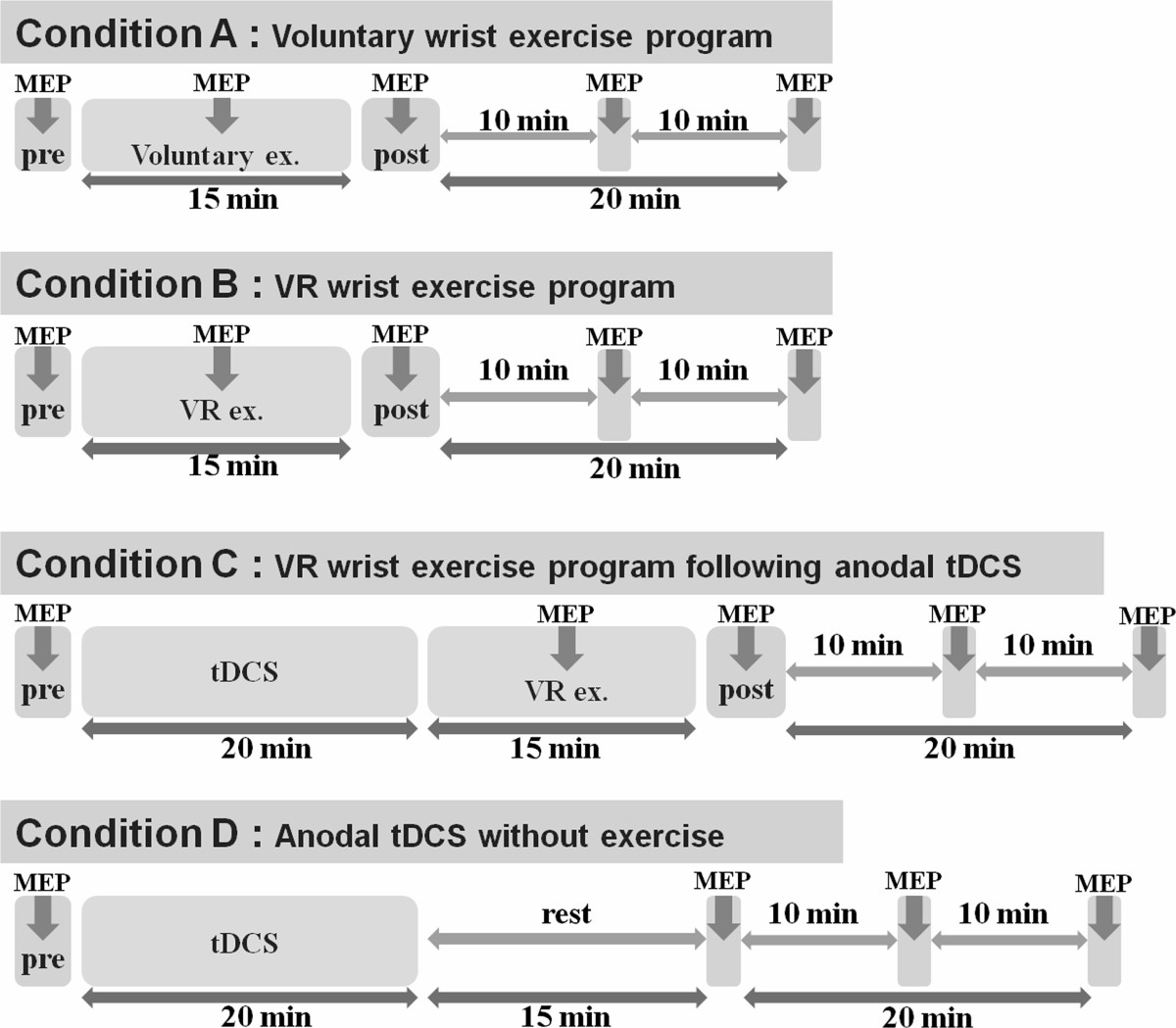
**Time points of the application of TMS.** A series of 12 TMS were applied repeatedly and we measured pre-, during- and post –exercise MEPs in each condition (**A,B,C**; thick arrow). In condition **D**, we measured pre- and post-tDCS MEPs without exercise. TMS, transcranial magnetic stimulation, MEPs, motor evoked potentials, VR, virtual reality, tDCS, transcranial direct current stimulation.

Subjects were seated in a comfortable chair, with head and armrests. TMS was performed with a 14-cm outer diameter circular coil attached to a MagPro R30 stimulator (MagVenture Inc., Farum, Denmark) and EMGs were measured using the Medelec Synergy EMG system (Natus Medical Inc., San Carlos, CA, USA). TMS was applied at the hot spot of the non-dominant primary motor cortex (M1) in healthy volunteers and the affected M1 area in stroke patients. The resting motor threshold (RMT) and the position and orientation of the coil were readjusted daily before the start of the experiment. Coil was held on the head by the experimenter. By marking the hot spot on a swimming cap, the position and orientation of the coil could be maintained throughout the course of the experiment and checked by experimenter. MEPs in the left (healthy) or affected (stroke) ECR were recorded using surface Ag/AgCl electrodes, 5 mm in diameter, as a measure of corticospinal excitability. The active and reference electrodes were attached to the motor point of the ECR, and tendon of the corresponding muscle. The ECR muscle was selected as the target muscle for facilitation because it plays a major role in our exercise program. EMG signals were amplified, filtered (10 Hz to 1 kHz), and then sampled at 5 kHz. Data were stored on a computer for off-line analyses. The RMT was determined as the minimum stimulation intensity required to evoke MEPs of more than 50 μV during at least 5/10 trials. The stimulation intensity was determined as 120% of RMT and was used consistently during each experiment. In each set of conditions (A, B, C), a series of 12 single TMSs was applied every 5 s and we measured pre-, during- and post-exercise (or post-tDCS) MEPs repeatedly (immediately, 10 min, 20 min). In condition D, we measured pre- and post-tDCS MEPs without exercise. Time points of the application of TMS are summarized in Figure 2. During recording, the left or affected side ECR muscle with the attached recording electrode was completely relaxed except during the exercise condition and muscle contraction was monitored in the EMG system. During the exercise condition, 12 TMSs were delivered during the extension phase of wrist movement, corresponding to a wrist joint angle of 60° during every flexion-extension cycle (inter-stimulus interval of 10 s) [36]. The wrist joint angle of 60° at the time of TMS delivery was confirmed with a goniometer printed on the desk.

### Attention and fatigue scores

After the intervention, the subjects rated their attention and fatigue in each condition on a visual analog scale (VAS; attention VAS, from 1: ‘no attention’ to 7: ‘highest level of attention’ and fatigue VAS, from 1: ‘no fatigue’ to 7: ‘highest level of fatigue’).

### Adverse events

We monitored adverse events during and after each session of stimulation and performed follow-up, monitoring whether subjects experienced any adverse event, and assessed the relationship of these events to the application of tDCS and TMS.

### Statistics

Peak-to-peak amplitudes of the 12 MEPs were recorded in each condition and the data were used to calculate mean values. Individual mean values were normalized to a percentage of the resting MEP for each subject, because the values of the MEPs were not normally distributed (Table 2). For statistical analyses, a linear mixed model for a repeated-measures covariance pattern model with unstructured covariance within groups (healthy or stroke groups) was used. Two fixed effects were included: between-subjects (A, B, C, D conditions in healthy volunteers and B, C, D conditions in stroke patients) and within-subject (time point: immediate, 10 min, 20 min after exercise). *Post hoc* analyses were also used to compare the conditions with each other using the Bonferroni correction for multiple comparisons when significant interactions of type of conditions and time existed. Repeated-measures ANOVA were used to compare task speed, distance, and the attention and fatigue scales among the three conditions. If there were significant differences among the three conditions, the Bonferroni multiple comparisons test was used. The paired *t*-test was used to compare the rate of coin gain, the fatigue scale, and the attention scale between the two conditions. The null hypothesis of no difference was rejected if *P* values were < 0.05. All data were analyzed using the SPSS software (ver. 18; SPSS Inc., Chicago, IL, USA) or the SAS software (ver. 9.2; SAS Inc., Cary, NC, USA).

**Table 2:** **Mean baseline MEP amplitude and RMT of each experiment between conditions**

	Healthy volunteers		Stroke patients	
Conditions	RMT (%)	MEP (μV)	RMT (%)	MEP (μV)
**Active wrist exercise**	50.9 ± 7.1	759.4 ± 231.2	N.A	N.A.-
**VR wrist exercise**	50.93 ± 8.1	660.8 ± 206.1	52.3 ± 7.7	494.25 ± 58.7
**VR wrist exercise following tDCS**	50.3 ± 8.3	651.7 ± 115.3	54.3 ± 6.2	483.9 ± 92.8
**tDCS only**	50.3 ± 9.1	594.5 ± 115.1	52.4 ± 6.7	472.7 ± 60.4

Values are given as means ± standard deviation (SD). RMT, resting motor threshold, MEP, motor evoked potential; NA, not available.

## Results

### Comparison of baseline MEPs and performance metrics among conditions

Mean baseline MEP amplitudes in each experiment did not show major differences among the A, B, and C conditions in healthy volunteers, and the B and C conditions in stroke patients (F_3,56_ = 2.297, *P* = 0.09; F_2,42_ = 0.333, *P* = 0.72; Table 2). Additionally, the mean baseline RMT in each experiment showed no major differences among conditions (F_3,56_ = 0.027, P = 0.99 in healthy volunteers; F_2,42_ = 0.394, *P* = 0.67 in stroke patients; Table 2).

Task speed and distance during exercise were compared among conditions in healthy volunteers and in stroke patients. There was no significant difference in task speed or distance among the A, B, and C conditions in healthy volunteers (F_2,42_ = 2.395, *P* = 0.10; F_2,42_ = 2.563, *P* = 0.09; Table 3), or B and C conditions in stroke patients (t = 1.205, *P* = 0.26; t = 1.303, *P* = 0.23; Table 3).

**Table 3:** **Comparison of performance metrics between conditions**

		Active wrist exercise	VR wrist exercise	VR wrist exercise following tDCS	***P***
Average task speed (°/s)	Healthy	32.9 ± 10.8	24.9 ± 3.6	31.9 ± 15.2	0.10
	Stroke	N.A.	17.3 ± 5.0	20.1 ± 10.8	0.26
Average distance (°)	Healthy	2010.7 ± 650.7	1499.9 ± 212.9	1920.7 ± 914.6	0.09
	Stroke	N.A.	1057.5 ± 279.6	1238.3 ± 628.9	0.23

P values from ANOVA and *t-*test. Values are means ± SD. VR, virtual reality, tDCS, transcranial direct current stimulation; NA, not available.

### Facilitation of corticospinal excitability in healthy volunteers

**Figure 3:**
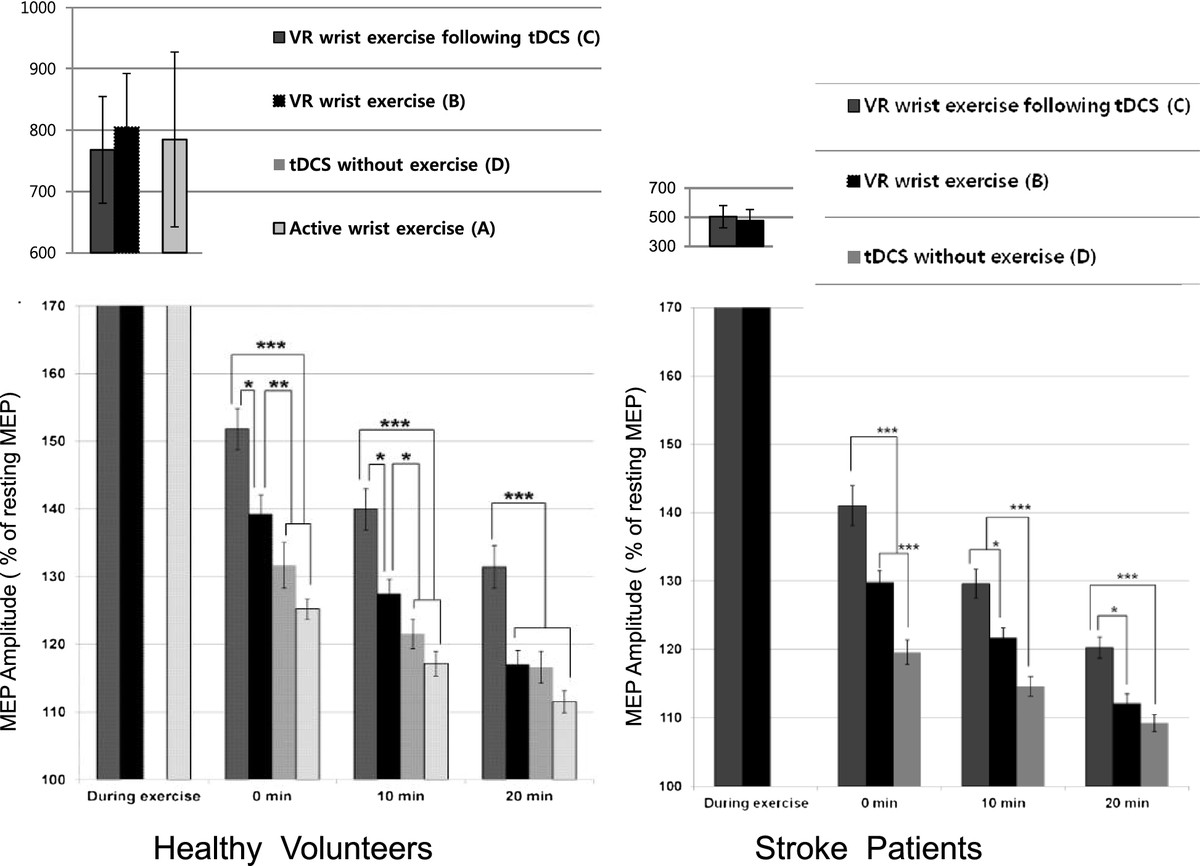
**Facilitation of corticospinal excitability.** The increase in MEP amplitude after VR wrist exercise following tDCS **(A)** was greater and sustained for 20 min after exercise compared with the three other conditions **(B,C,D)** in healthy volunteers and other two conditions **(B,C)** in stroke patients (***P < 0.001, **P < 0.01, *P < 0.05). *P* value from the *post hoc* analysis with Bonferroni correction. Error bars indicate standard errors of the mean (SEM). MEPs, motor evoked potentials, VR, virtual reality, tDCS, transcranial direct current stimulation.

MEP facilitation during exercise did not show any difference among conditions A, B, and C in the healthy volunteers (F_2,43_ = 0.05, *P* = 0.95). However, there were immediate increases in percentage MEP amplitude (% amplitude at rest) after exercise in conditions A, B, and C and after application of anodal tDCS without exercise (Table 4). There was a significant main effect between the four conditions and the three time points (*F*_*6,84*_ = 5.48; *P* < 0 .001). *Post hoc* comparisons showed that the immediate increase in the MEP amplitude after VR exercise following tDCS was greater than that in the other conditions A, B, and D (A: t = 7.69, *P* < 0.001; B: t = 3.24, *P =* 0.01; D: t = 5.79, *P* < 0.001; Figure 3). The immediate increase in MEP amplitude after VR exercise was greater than that for either the active wrist exercise (A) or the tDCS without exercise condition (D) (A: t = 4.91, *P* < 0.001; D: t = 3.62, *P* = 0.003; Figure 3). The immediate increase in MEP amplitude after active wrist exercise showed no difference versus tDCS without exercise (t = 2.42, *P* = 0.10). These differences in MEP amplitudes were sustained for 10 min after exercise. After 20 min, the MEP amplitude was decreased and there was no difference between the VR wrist exercise and active wrist exercise (A) or tDCS without exercise conditions (D) (A: *P* = 0.41, D: *P* = 4.9; Table 4). However, the facilitated amplitude of VR wrist exercise following anodal tDCS was sustained for 20 min after exercise versus the other three conditions (A, B, D: *P <* 0.001; Figure 3).

**Table 4:** **Facilitation of corticospinal excitability between four conditions**

Conditions		Rest	During exercise	Immediate after exercise or tDCS	10 min after exercise	20 min after exercise
Active wrist exercise (A)	Healthy	100	785 ± 553	125 ± 6	117 ± 7	112 ± 6
VR wrist exercise (B)	Healthy	100	805 ± 340	139 ± 11	127 ± 8	117 ± 8
	Stroke	100	475 ± 297	130 ± 7	122 ± 6	112 ± 5
VR wrist exercise following tDCS (C)	Healthy	100	768 ± 337	152 ± 12	140 ± 12	131 ± 12
	Stroke	100	502 ± 297	141 ± 11	130 ± 8	120 ± 6
tDCS without exercise (D)	Healthy	100	-	132 ± 13	121 ± 8	117 ± 9
	Stroke	100	-	120 ± 7	115 ± 6	109 ± 5
Overall P value	Healthy	Condition: <0.001, time: <0.001, condition X time: <0.001				
	Stroke	Condition: <0.001, time: <0.001, condition X time: <0.001				
C Vs A	Healthy	-	-	<0.001	<0.001	<0.001
C Vs B*	Healthy	-	-	0.01	0.01	<0.001
	Stroke	-	-	<0.001	0.01	0.01
C Vs D*	Healthy	-	-	<0.001	<0.001	<0.001
	Stroke	-	-	<0.001	<0.001	<0.001
B Vs A*	Healthy	-	-	<0.001	0.002	0.41
B Vs D*	Healthy	-	-	0.003	0.03	4.96
	Stroke	-	-	<0.001	<0.001	0.14
A Vs D*	Healthy	-	-	0.10	0.63	0.36

Values were transformed into a percentage of the MEP at rest. Values are mean ± SD.

*Post hoc P values were corrected by Bonferroni method.

VR, virtual reality, tDCS, transcranial direct current stimulation, −, not available.

### Facilitation of corticospinal excitability in stroke patients

MEP facilitation during exercise showed no difference between VR wrist exercise alone and VR exercise following tDCS in stroke patients (t = 0.96, *P* = 0.35). Likewise, in healthy volunteers, there was an immediate increase in the percentage MEP amplitude (% amplitude at rest) after the VR exercise in the B and C conditions or after the application of anodal tDCS without exercise (Table 4). We found a significant main effect of the three conditions (B, C, and D) and the three time points (*F*_*4,56*_ = 16.99; *P* < 0.001). *Post hoc* comparisons showed an immediate increase in MEP amplitude after VR exercise following tDCS that was greater than with VR wrist exercise alone (t = 7.64, *P <* 0.001: Figure 3). The immediate increase in MEP amplitude after VR exercise was greater than in tDCS without exercise (t = 7.17, *P* < 0.001; Figure 3). These differences in MEP amplitudes were sustained for 10 min after exercise. After 20 min, the MEP amplitudes had decreased and there was no difference between VR exercise alone and tDCS without exercise (t = 2.03, *P* = 0.14; Table 4). However, the increase in amplitude in the VR wrist exercise following anodal tDCS was sustained for 20 min after exercise versus conditions B and D (B: t = 3.06, *P* = 0.01; D: t = 3.91, *P <* 0.001; Figure 3).

### Comparison of rate of coin acquisitions (VR exercise alone vs. VR exercise combined with tDCS)

The mean number of achieved coin per one minute revealed in healthy volunteers was 14.9 ± 1.2 (VR condition) and 15.2 ± 0.7 (VR-tDCS condition). In stroke patients, they were 9.5 ± 3.0 (VR condition) and 9.3 ± 2.68 (VR-tDCS condition). We compared the performance score across two VR exercise conditions (VR vs. VR-tDCS). There were no significant differences in the rate of coin acquisitions between the two conditions in healthy volunteers or stroke patients (93.7 ± 7.7 vs. 94.7 ± 4.3 in healthy volunteers; t = 1.30, *P* = 0.221, 78.1 ± 18.9 vs. 72.6 ± 24.3 in stroke patients; t = 0.57, *P* = 0.585).

### Attention and fatigue scales between conditions in healthy and stroke subjects

**Figure 4:**
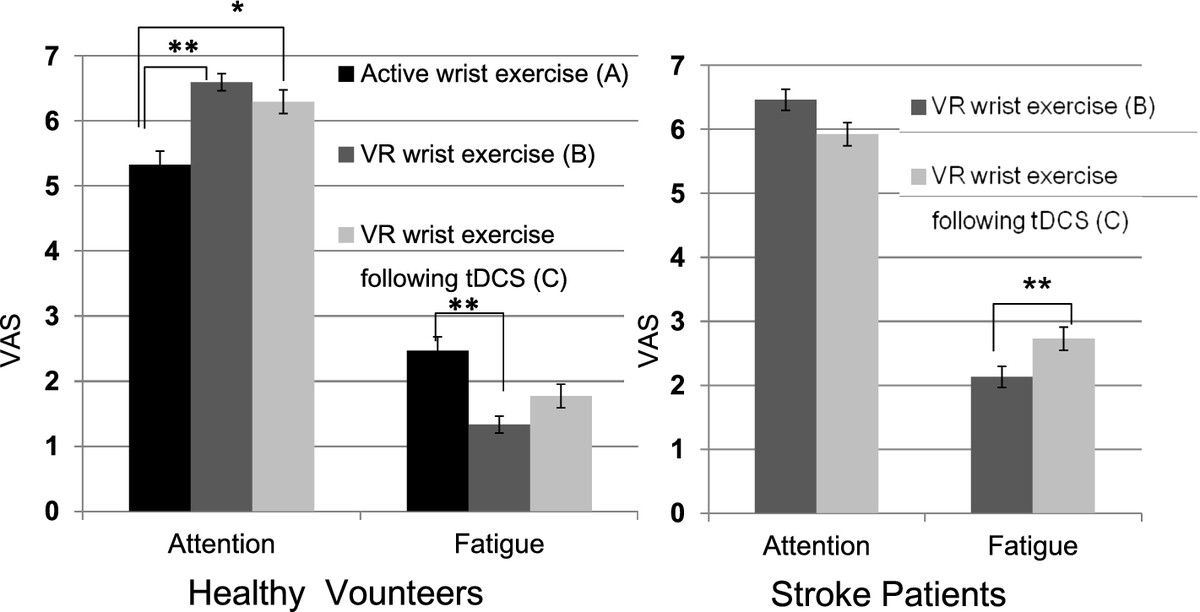
**Attention and fatigue scales between conditions.** Level of attention and fatigue was presented by VAS, from 1: ‘no attention’ to 7: ‘highest level of attention’ and from 1: ‘no fatigue’ to 7: ‘highest level of fatigue’**.** Error bars indicate SEM. P values were from ANOVA and t-test (**P < 0.01, *P < 0.05). VR: virtual reality, tDCS: transcranial direct current stimulation, VAS: visual analog scale.

We measured attention and fatigue using VASs. In healthy volunteers, the average attention scale ratings were 5.3 ± 0.8 (condition A), 6.6 ± 0.5 (condition B), and 6.3 ± 0.7 (condition C). In stroke patients, the attention scale ratings were 6.5 ± 0.6 (condition B) and 5.9 ± 0.7 (condition C). The average fatigue scale ratings were 2.5 ± 0.9 (condition A), 1.3 ± 0.6 (condition B), and 1.5 ± 0.5 (condition C) in healthy volunteers. In stroke patients, the average fatigue scale ratings were 2.1 ± 0.4 (condition B), and 2.7 ± 0.7 (condition C). In healthy volunteers, there were significant differences in attention (ANOVA; F_2,42_ = 10.2, *P* = 0.002) and fatigue scores among the three conditions (ANOVA; F_2,42_ = 9.39, *P* = 0.001). *Post hoc* analysis revealed that VR wrist exercise alone showed significantly higher attention and lower fatigue scores than active wrist exercise in healthy volunteers (*P* < 0.01, Figure 4). VR wrist exercise alone showed lower fatigue scores (t = 3.15, *P* = 0.007) and higher attention scores (t = 2.08, *P* = 0.05) compared with VR exercise following tDCS in stroke patients (Figure 4).

### Adverse effects

All participants tolerated TMS, tDCS, and VR well without experiencing significant adverse effects. The few reported adverse events were all mild, and consisted of mild headache (in one stroke patient) and itching under the electrodes (in one stroke patient and three healthy volunteers).

## Discussion

These results showed that VR wrist exercise following tDCS had greater immediate and sustained post-exercise corticospinal facilitation effects than exercise without tDCS or than tDCS without exercise. Furthermore, post-exercise corticospinal facilitation was sustained for 20 min after exercise in the VR and tDCS conditions compared to the other conditions.

### VR training-induced increase in cortical excitability

In the present study, VR motor training facilitated post-exercise corticospinal excitability more than simple active exercise in healthy volunteers. Although not consistent, most previous studies revealed that repeated motor training increased motor cortex excitability immediately and reduced intra-cortical inhibition [37–40]. However, the type of motor training is crucial for the post-exercise facilitatory effects on the motor cortex [41, 42]. In this study, we compared the post-exercise facilitatory effects between VR wrist exercise paradigm and active exercise. Short-term changes in corticospinal excitability after visuomotor adaptation using a VR program were assessed as a marker for learning-related processes; these facilitatory effects might accelerate motor recovery in stroke patients [15]. These changes in M1 excitability may lead to sustained, cumulative changes, and are associated with motor learning and better post-stroke clinical outcomes [40, 43–45].

The detection rate of wrist movements was about 30 Hz in our VR system, a rate that was sufficient for examining wrist movements that were performed one cycle about every 6 s. This was much slower than 30 Hz, so the wrist movements were fully detectable, although conventional movement measuring systems have much higher recording rates [46, 47].

To the best of our knowledge, this is the first report to show that VR wrist exercise facilitates post-exercise corticospinal excitability more than a paced- active wrist exercise (Figure 3; A, B). Comparing VR to the paced- active wrist exercise, there was no significant difference in duration of exercise (15 min), performance metrics (movement speed, total distance), or MEP facilitation during exercise. This suggests that the superior facilitation by VR exercise resulted from factors other than differences in muscle activation during exercise. One possible reason for the enhanced post-exercise facilitation of corticospinal activity by the VR exercise could be found in the characteristics of VR exercise, which is task-oriented (catching coins and successful jumping), more interactive, and more interesting. Thus, it draws subject attention that might activate the ipsilesional extended motor network, including a putative mirror neuron system [48].

The present study and many previous findings support our interpretation. We found that VR wrist exercise produced a higher level of attention and a lower fatigue scale score than active wrist exercise (Figure 4). It is known that the type of motor activation is important for the occurrence of the post-training facilitatory effects of MEP [41, 42]. Perez et al. showed that repetitive motor skill exercises but not non-skill motor passive exercises, increased post-exercise corticospinal excitability evoked by TMS [37]. They also observed a decrease in intra-cortical inhibition after motor skill exercises and explained the increase in MEPs as due to possible modulation by local intra-cortical circuits [37]. These findings indicate that observed changes in activities at the cortical level may be related to the type of motor activity, degree of attention and fatigue, and goal-directed behavior in the motor tasks [37, 41, 49, 50].

Another possible explanation also supports the idea that coordination between visual input and motor performance is the decisive factor for the VR exercise-induced changes in cortical excitability. Visuo-motor training similar to that used in the present study increased activity in cortical neurons in monkeys and can improve motor performance in humans [27, 49, 51].

### Combined tDCS and VR wrist exercise-induced increases in cortical excitability

Nitsche and Paulus noted that up to 40% of MEP excitability changes appeared and lasted for several minutes after the end of anodal tDCS in healthy volunteers [17]. Additionally, anodal tDCS increased the MEPs of affected muscle in patients with stroke and healthy subjects [52]. These facilitatory effects are believed to accelerate motor recovery in stroke patients [53]. The strength and duration of these after-effects could be controlled by varying the intensity and duration of anodal tDCS [17]. In this study, after 20 min of tDCS, the MEP returned to baseline after 20 min of no exercise. However, if VR exercise was performed immediately after tDCS, the corticospinal excitability was sustained for another 20 min.

A possible mechanism for the increased duration is the cortical excitability effect of anodal tDCS, VR exercise induced corticospinal facilitation, and the VR exercise-induced decrease in cortico-cortical inhibition may act synergistically. Although we did not assess intracortical inhibition, many previous studies have demonstrated a decrease in intracortical inhibition during and after skill-acquisitive voluntary motor training or tDCS [40, 54–56]. Reduced intracortical inhibition is important for inducing neural plasticity after injury [40, 55, 57]. However, in present study, VR exercise combined with tDCS did not increase or decrease the subjects’ rated attention or fatigue scores versus those in VR exercise alone.

Few studies have assessed the synergistic effect of tDCS and motor skill training. Two studies reported a beneficial effect of combined peripheral nerve stimulation and tDCS on motor sequence performance in chronic stroke patients [58], and increased corticomotor excitability of the motor cortex that persisted after anodal tDCS during robotic wrist training [56]. One recent randomized multicenter trial noted no significant functional improvement in robot arm training during tDCS in 96 stroke patients [5]. Considering that this study enrolled mostly patients whose upper extremities were severely impaired (FMS < 10) and had cortical lesions, unlike our study, further studies are needed to address its effects.

In this study, a single session of VR exercise following tDCS did not produce a higher score for the rate of coin acquisitions than VR exercise alone. One reason might be that tDCS was performed before the task in the present study. While our study did not show that a single session of combined therapy (tDCS and VR) would improve motor performance more than single therapy (VR alone), the rationale for this combined therapy is that cortical facilitation across multiple practice sessions may translate into enhanced and sustained neuroplastic changes in the affected hemisphere [40]. Several studies have demonstrated long-lasting learning effects or functional improvement of skilled motor training following multiple sessions of tDCS in healthy volunteers and stroke patients [21, 33, 34, 59].

There were several limitations to our study. First, this study was conducted using a small sample of mildly impaired stroke patients. All subacute stroke patients were in a period of spontaneous recovery. We believe the impact of this factor was minimized because we tested three tasks in randomized order and the test was completed over 3 or 4 days. Second, heterogeneity in type of lesion (cortical or subcortical) and side of stimulation could be other limitations. Subcortical stroke patients with intact cortical connectivity may profit more from tDCS than patients with disrupted neural pathways [5]. Furthermore, use-dependent cortical plasticity may differ according to the stimulated hemisphere, according to previous studies using TMS [27]. Thus, we analyzed hemisphere-specific facilitation in stroke patients, which showed comparable results. Third, we did not compare the full performance or behavioral measurement of the upper extremity (except rate of coin acquisition) among VR conditions with or without tDCS. We focused on the change in post-exercise corticospinal facilitation according to the various exercise conditions, especially the synergistic effect of tDCS and VR conditions. Fourth, a lack of sham stimulation or multiple mode simulation of tDCS (e.g., dual hemisphere stimulation [60]) was another limitation. The mode, repetition, and duration of stimulation of tDCS, anatomical location of lesions, grade of severity of impairment, and type of training could affect the cortical facilitation of tDCS after stroke. Future studies should investigate whether the simultaneous application of tDCS and exercise may induce greater behavioral changes, and these results should be compared to those from the present study.

## Conclusions

We report that VR motor training facilitated corticospinal excitability after exercise more than simple active exercise in healthy volunteers. Furthermore, the combined effect of VR motor training following tDCS was synergistic and short-term corticospinal facilitation was superior to the application of VR training, active wrist motor training, or tDCS without exercise in healthy volunteers and subacute stroke patients. These results support the concept of combining brain stimulation with VR motor training to promote recovery after stroke. Further work is also warranted to investigate functional improvement after combined training in larger patient groups and optimal application of this combined treatment, in terms of mode, method of stimulation, and various types of training.

## Authors’ information

Dr Kang (YJK) is an associate professor in the Rehabilitation Department at Eulji Hospital, Eulji University School of Medicine. Her research focuses on rehabilitation of stroke patients using virtual reality or other novel technologies. Dr. Kang has authored several recent publications, including “Upper extremity rehabilitation of stroke: Facilitation of corticospinal excitability using virtual mirror paradigm**”** (First author, J NeuroEng Rehab, Oct 4, 2012), “Development of virtual reality proprioceptive rehabilitation system for stroke patients” (Corresponding author***,*** Computer Methods and Programs in Biomedicine 2014:113;258–265), “Facilitation of corticospinal excitability according to motor imagery and mirror therapy in healthy subjects and stroke patients” (First author, Ann Rehabil Med 2011;35, 747–758), and “Validity and Reliability of Cognitive Assessment Using Virtual Environment Technology in Patients with Stroke.” (Corresponding author, Am J Phys Med Rehabil 2009;88:702–710).

## Authors’ original submitted files for images

Below are the links to the authors’ original submitted files for images. Authors’ original file for figure 1 Authors’ original file for figure 2 Authors’ original file for figure 3 Authors’ original file for figure 4

## Abbreviations

VR: Virtual reality
tDCS: Transcranial direct current stimulation
MEP: Motor evoked potentiall
TMS: Transcranial magnetic stimulation.
