# Supplementary figures and images for: Facilitation of corticospinal excitability by virtual reality exercise following anodal transcranial direct current stimulation in healthy volunteers and subacute stroke subjects
Source: J Neuroeng Rehabil. 2014 Aug 18;11:124. doi: 10.1186/1743-0003-11-124 (PMC4148539; doi:10.1186/1743-0003-11-124)

A

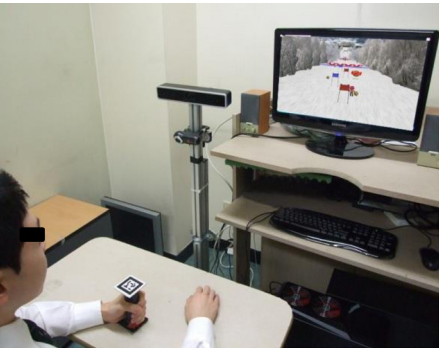

B

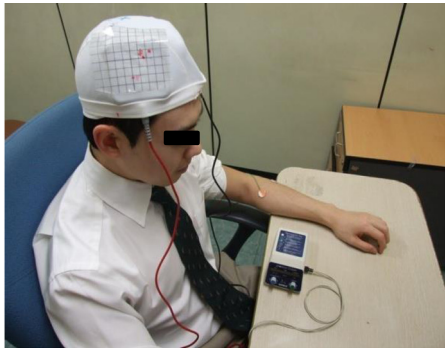

Supplement: Supplementary file 1 — Authors’ original file for figure 1 [file 12984_2014_646_MOESM1_ESM.pdf]

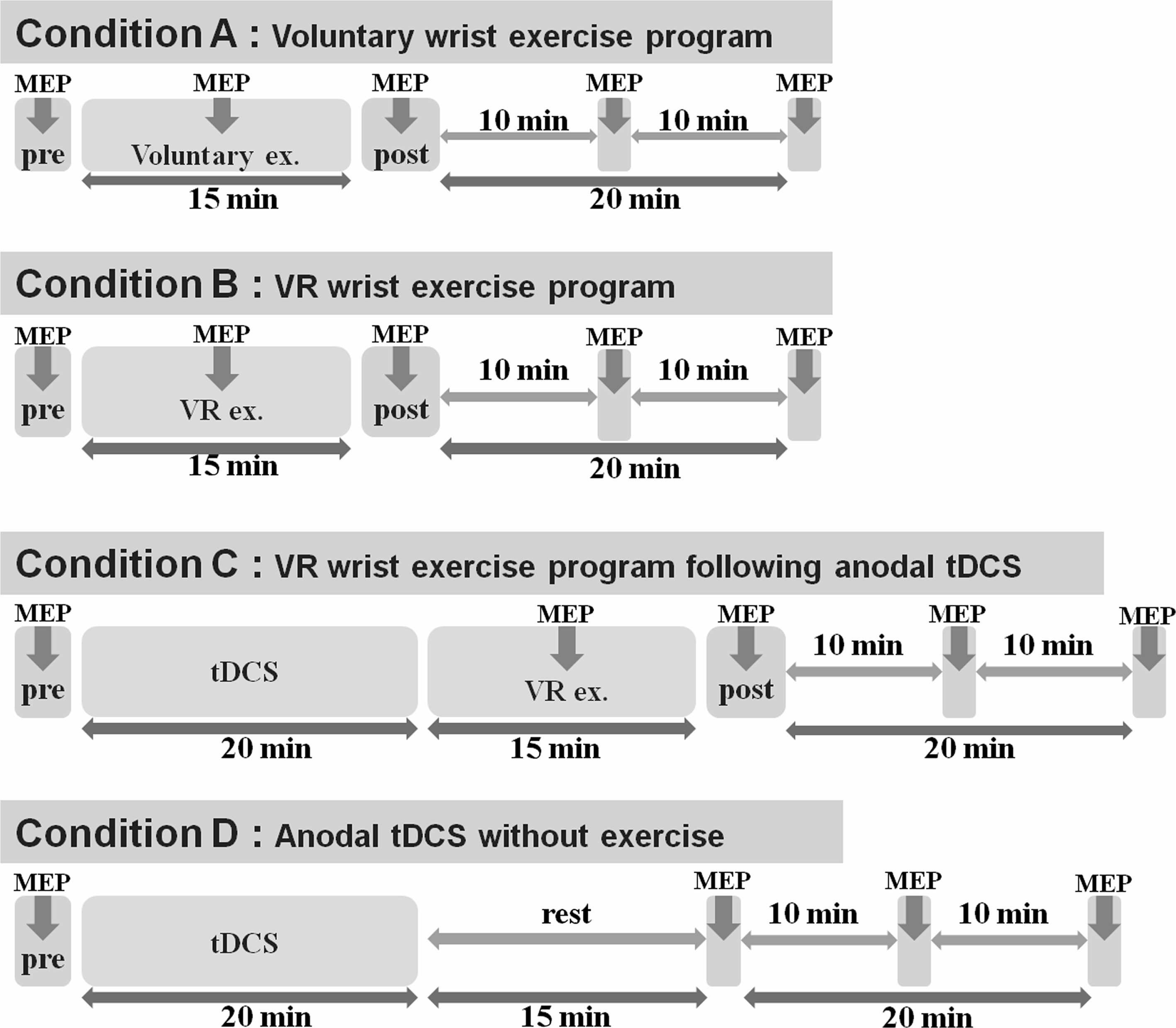

Supplement: Supplementary file 2 — Authors’ original file for figure 2 [file 12984_2014_646_MOESM2_ESM.tiff]

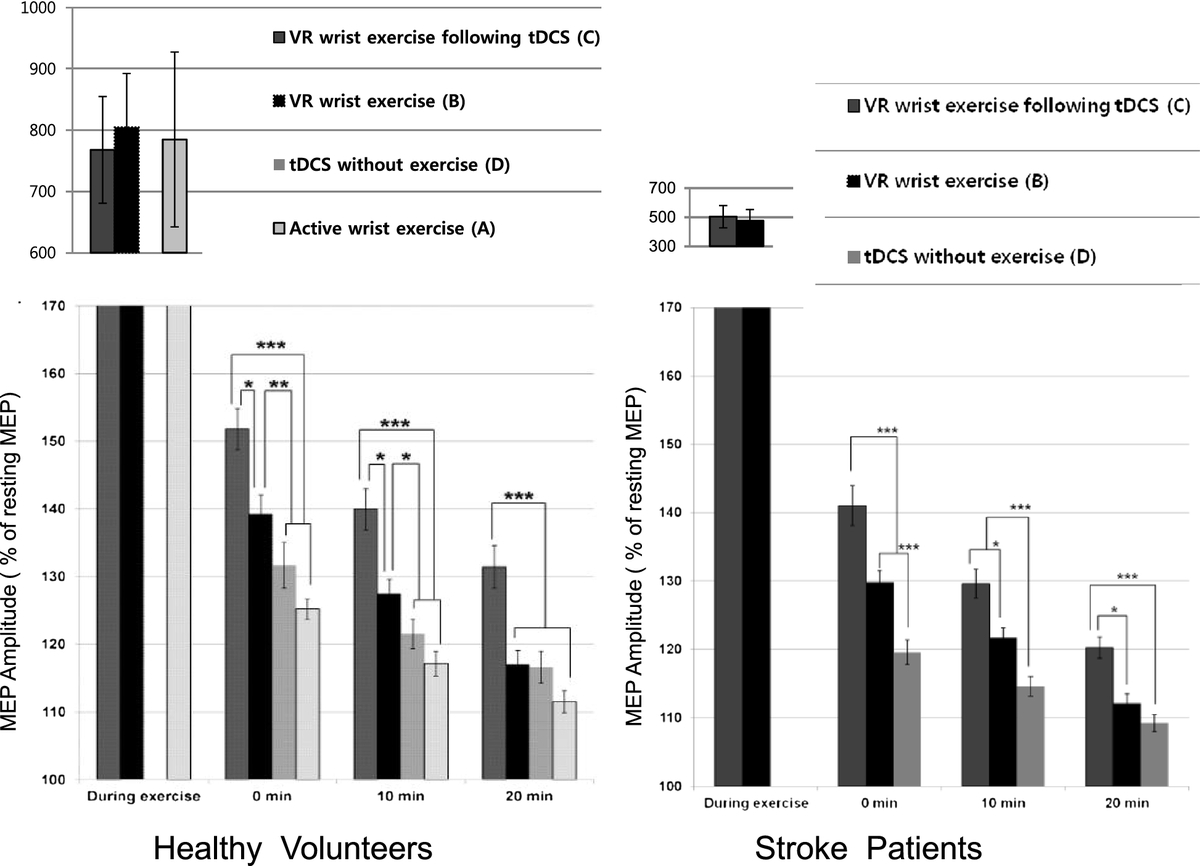

Supplement: Supplementary file 3 — Authors’ original file for figure 3 [file 12984_2014_646_MOESM3_ESM.jpeg]

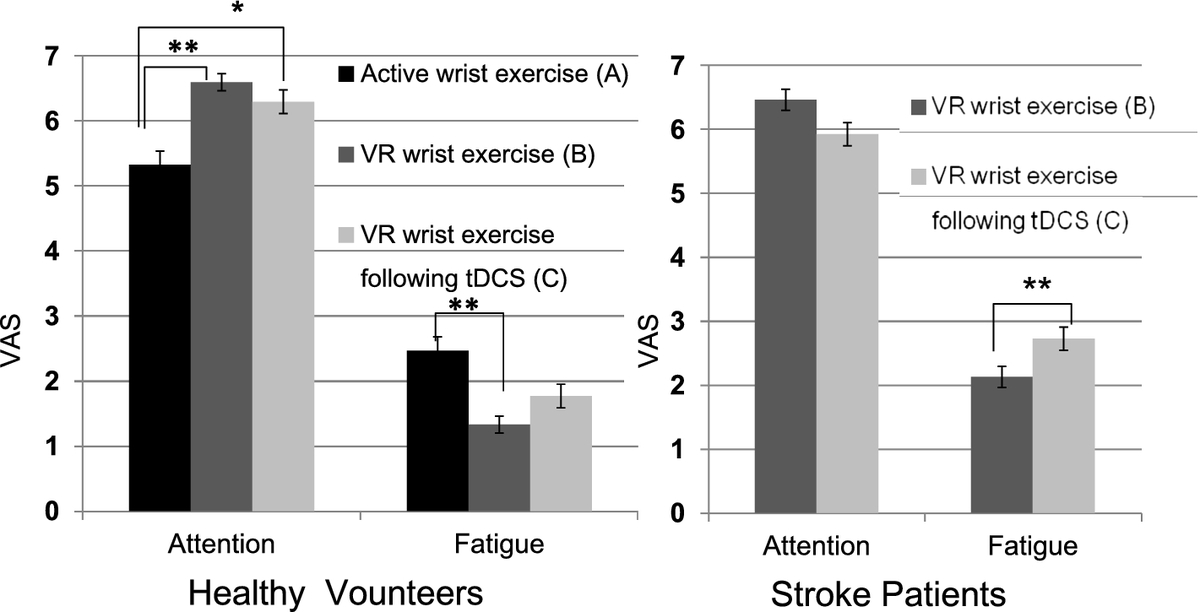

Supplement: Supplementary file 4 — Authors’ original file for figure 4 [file 12984_2014_646_MOESM4_ESM.jpeg]
